# Distinguishing Cardiac Amyloidosis and Hypertrophic Cardiomyopathy by Thickness and Myocardial Deformation of the Right Ventricle

**DOI:** 10.1155/2022/4364279

**Published:** 2022-02-01

**Authors:** Hui Liu, Peng Bai, Hua-Yan Xu, Zhen-Lin Li, Chun-Chao Xia, Xiao-Yue Zhou, Liang-Geng Gong, Ying-Kun Guo

**Affiliations:** ^1^Department of Radiology, Second Affiliated Hospital of Nanchang University, Nanchang 330000, China; ^2^Department of Forensic Genetics, West China School of Basic Medical Sciences & Forensic Medicine, Sichuan University, Chengdu 610041, Sichuan, China; ^3^Department of Radiology, Key Laboratory of Obstetric & Gynecologic and Pediatric Diseases and Birth Defects of Ministry of Education; West China Second University Hospital, Sichuan University, Chengdu, Sichuan 610041, China; ^4^Department of Radiology, National Key Laboratory of Biotherapy, West China Hospital, Sichuan University, Chengdu, Sichuan 610041, China; ^5^MR Collaboration, Siemens Healthcare Ltd., Shanghai, China

## Abstract

**Objectives:**

To compare right ventricular thickness (RVT) and deformation of cardiac amyloidosis (CA) and hypertrophic cardiomyopathy (HCM) patients.

**Methods:**

Sixty CA (mean age 58 ± 10 years; 33 males (55%)) and sixty HCM patients (mean age 55 ± 14 years; 27 males (45%)) were retrospectively enrolled. RVT, global radical peak strain (GRPS), global longitudinal peak strain (GLPS), and global circumferential peak stain (GCPS) were analyzed. To determine the cutoff values of the RVT and RV strain parameters for distinguishing CA from HCM, the areas under the receiver operating characteristic curve (AUCs) were analyzed.

**Results:**

RVT of CA patients was significantly thicker than that of HCM patients (7.8 ± 2.1 vs 5.9 ± 1.3, *p* < 0.001). Moreover, significantly decreased RV-GRPS (12.1 ± 6.9 vs 23.5 ± 12.1, *p* < 0.001), RV-GCPS (−3.4 ± 2.2 vs −5.6 ± 3.5, *p* < 0.001), and RV-GLPS (−4.6 ± 2.3 vs −11.1 ± 4.9, *p* < 0.001) were observed in CA patients compared with HCM patients. RVT and RV strain demonstrate comparable diagnostic accuracy in differentiating CA from HCM. In particular, RV-GLPS combined with RVT showed the best performance for discriminating CA from HCM (AUC = 0.92, 95% CI: 0.85 to 0.96, *p* = 0.0001).

**Conclusions:**

Right ventricular myocardial thickness and deformation of CA patients was more severe than HCM patients. RV-GLPS combined with RVT presents an excellent diagnostic performance in distinguishing CA and HCM.

## 1. Introduction

Cardiac amyloidosis (CA) is defined by the presence of extracellular amyloid deposition within the myocardium of the whole heart, leading to biventricular wall thickening with impaired relaxation and the loss of ventricular elasticity [1]. Due to the ventricular hypertrophy caused by amyloid deposition, CA has often been misdiagnosed as hypertrophic cardiomyopathy (HCM), which has main macroscopic characteristics of myocardial wall thickening and myocyte hypertrophy [2, 3]. Clinically, the differentiation of CA from HCM is extremely important owing to the diverse therapeutic options and difference in long-term prognosis.

As an important differential diagnostic index, the structure and function of the left ventricle (LV) has been identified and shown to discriminate between CA and HCM to a certain extant [4–6]. Though right ventricle thickness (RVT) is extensively involved in CA but less in HCM [7], the differences in RVT and RV deformation were underestimated. Due to the high spatial resolution and difficult acoustic windows, cardiovascular magnetic resonance (CMR) imaging is now considered the gold standard technique for RV morphological study. Moreover, CMR tissue tracking (TT) technique, which could measure cardiac muscle motion and both LV and RV deformation, has emerged as more sensitive indicators than the ejection fraction (EF) [8–12]. Thus, the aims of this study were as follows: (1) to assess and compare RV thickness (RVT) and RV deformation parameters derived from the CMR-TT technique between CA and HCM patients and (2) to further identify the most valuable RV parameters for differentiating CA from HCM.

## 2. Methods

### 2.1. Study Population

This study was approved by our institutional review board. We retrospectively studied 63 patients with CA (mean age 58 ± 11 years; range 25–81 years, 35 males [56%]) from 2015 to 2019. All patients were diagnosed with light chain amyloidosis. A diagnosis of light chain amyloidosis was made based on a biopsy of subcutaneous fat or an involved organ with the demonstration of typical Congo red birefringence, the detection of a monoclonal protein in the serum or urine and/or a monoclonal population of plasma cells in the bone marrow [13]. Furthermore, the diagnosis criteria of CA were based on a consensus opinion from the 10th International Symposium on Amyloid and Amyloidosis: LV wall thickness >12 mm without another known cause, as shown by echocardiography or cardiovascular magnetic resonance imaging [14]. Other than the bone marrow, the other tissue specimens for biopsy were obtained from the kidney (*n* = 13, 22%), liver (*n* = 1, 2%), and fat (*n* = 3, 5%). The exclusion criteria included (1) congenital heart disease (*n* = 0); (2) coronary artery disease (*n* = 1); (3) severe arrhythmia (*n* = 1); and (4) poor quality CMR images (*n* = 1). Finally, 60 CA patients (mean age 58 ± 10 years; range 25–81 years, 33 males [55%]) were enrolled.

We further included 60 patients with HCM (mean age: 56 years, range: 18–83 years; 27 males [45%]). The diagnostic criteria for subjects with HCM were based on the guidelines from the European Society of Cardiology [15]. All HCM patients enrolled in our study had nonobstructive hypertrophic cardiomyopathy, which can be divided into the following categories according to the segments of hypertrophic myocardium: (1) interventricular septal hypertrophic (*n* = 39), (2) anterolateral wall hypertrophic (*n* = 11), (3) posterior wall hypertrophic (*n* = 4), and (4) diffuse LV hypertrophic (*n* = 6). All of the patients were matched to CA patients in terms of maximum thickness of the LV segments. A total of 30 age-matched healthy volunteers (mean age: 56 years, range: 24–80 years; 17 males [57%]) were enrolled as normal controls. The inclusion criteria for normal controls included no hypertension (blood pressure <140/90 mmHg), normal 12-lead electrocardiography (ECG), and no history or symptoms of cardiovascular disease or diabetes. All patients and normal controls underwent CMR imaging for morphologic and deformation analysis.

### 2.2. CMR Imaging Protocol

CMR was performed using a 3.0-T whole-body scanner (Magnetom Tim Trio; Siemens Healthineers, Erlangen, Germany) with an 18-element body phased-array coil and ECG-triggering device during breath holding. Steady-state free precession sequences were performed to acquire consecutive short-axis cines covering the LV from the mitral valve level to the apex in 8 slices (TR/TE 37.66 ms/1.2 ms, flip angle 39°, FOV 280 mm × 373 mm, matrix size 146 mm × 280 mm, slice thickness 8 mm), while two-chamber long-axis and four-chamber cine series were acquired using the same sequences.

### 2.3. Imaging Analysis

**Figure 1:**
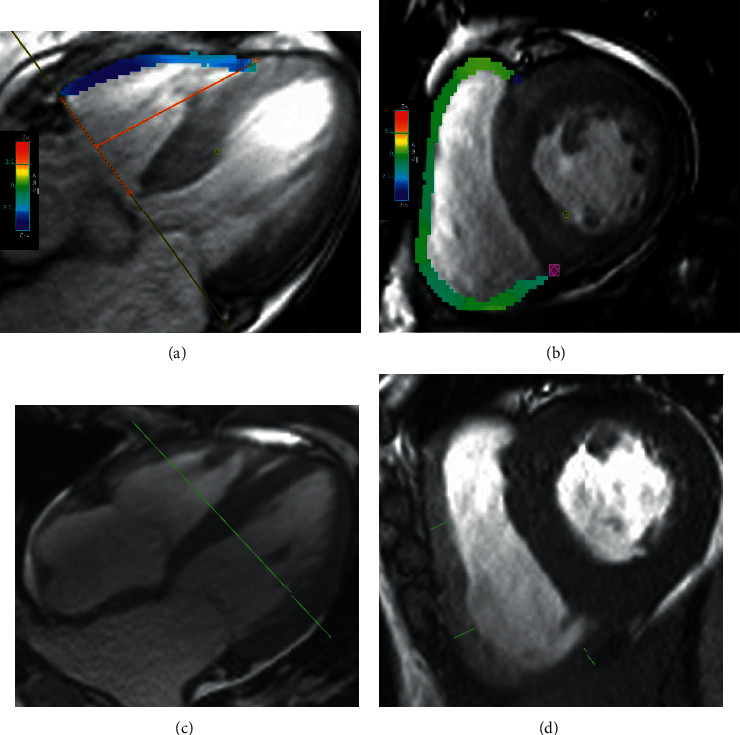
Diagrams of right ventricular (RV) strain analysis for global radial peak strain (GRPS), circumferential peak strain (GCPS), and longitudinal peak strain (GLPS). The four chambers (a) and short-axis (b) of the heart show a diagram for the RV strain. Measurement of the right ventricular thickness (RVT) of the inferior and anterior free wall (green line) of the midventricular from short-axis cine images (c-d) at the end of diastole.

All image analyses were performed using commercially available software (cvi42; Circle Cardiovascular Imaging, Inc. Calgary, Canada). To measure cardiac function, endocardial and epicardial traces were performed manually in serial short-axis slices at the end-diastolic and end-systolic phases. Global LV/RV systolic function, including LV/RV end-diastolic volume (EDV), end-systolic volume (ESV), and LV/RV ejection fraction, were computed. LV and RV myocardial strain analysis was performed by loading the long-axis four-chamber and short-axis slices into the tissue tracking module (Figures 1(a) and 1(b)). The RVT was determined three times of the midventricular, and the average thickness was calculated (Figures 1(c) and 1(d)). The global feature tracking parameters were acquired automatically, including global radical peak strain (GRPS), global longitudinal peak strain (GLPS), and global circumferential peak stain (GCPS). Peak systolic strain rate (PSSR, maximum strain rate in absolute value over all phases starting from diastole until the next systole) and peak diastolic strain rate (PDSR, maximum strain rate in absolute value over all phases starting from systole until the next diastole) were also analyzed. Positive and negative symbols represent different directions of motion. As previously described [16], a normal RVT value was defined as ≤7 mm. According to the criteria, we divided CA and HCM patients into subgroups according to patients with RV hypertrophy (RVT >7 mm) or without RV hypertrophy (RVT ≤7 mm).

### 2.4. Biomarkers

In CA and HCM patients, the blood was collected within two weeks of the CMR examination to measure myoglobin, creatine kinase isoenzyme, N-terminal pro-brain natriuretic peptide (NT-proBNP), cardiac troponin *T*, triglyceride (TG), and cholesterol (CHOL) levels.

### 2.5. Reproducibility Analysis

To verify the reproducibility and reliability of RV functional assessments by CMR in our cohort, intra- and interobserver variability were calculated for the RV strain parameter measurements in 15 randomly selected CA patients and 15 randomly selected HCM patients. For intraobserver variability, the parameters were measured twice by the same observer with a minimum interval of two weeks between serial assessments. Interobserver variability was assessed using measurements obtained by a second independent observer blinded to all other analyses. These values are presented as mean ± standard deviation (SD).

### 2.6. Statistics Analysis

All statistical analyses were performed using a commercially available software package (SPSS for Windows, version 25.0, SPSS Inc., Chicago, IL; GraphPad, version 7.00, GraphPad Software, Inc., La Jolla, CA, USA; and MedCalc, version 9.3.0.0., MedCalc Software, Mariakerke, Belgium). All data were evaluated for normal distributions using the Kolmogorov–Smirnov test. Homogeneity of variance was evaluated using Levene's test. The results are expressed as mean ± standard deviation (SD) or median (interquartile range [IQR], 25%–75%). An independent samples *t*-test and Mann–Whitney *U* test were used to evaluate the baseline and strain parameters of the subgroups. To determine the cutoff values of the different RV parameters for diagnosing CA among patients with increased wall thickness, receiver operating characteristic curves were constructed, and the Youden index was used. Multiple receiver operating characteristic curves were compared based on the methodology described by Delong et al. to determine the differential diagnostic capacity [17]. Inter- and intraobserver variability for the RV strain parameters were assessed in 30 patients with CA (*n* = 15) and HCM (*n* = 15) using the Bland–Altman method, the results of which were presented as percentage mean bias ± SD and 95% confidence interval (CI). A two-sided *p* value < 0.05 was considered statistically significant for all tests.

## 3. Results

### 3.1. Baseline Characteristics

Baseline characteristics and biomarkers are shown in Table 1. Among 60 CA patients, 3 patients (7%) had hypertension, and 2 (7%) had diabetes. Among the 60 HCM patients, 2 (3%) had hypertension, and 2 (3%) had diabetes. Patients with CA had lower systolic pressure than HCM patients (113 ± 18 vs 122 ± 15, *p* = 0.036). The laboratory tests showed that CA patients had significantly higher myoglobin (50.96 (34.46–80.04) vs 31.93 (25.04–45.55), *p* = 0.005), NT-proBNP (5803.5 (2533–13969) vs 1513 (656–3516), *p* = 0.001), and troponin *T* (91.9 (53.85–223.45) vs 17.3 (11.9–45.3), *p* = 0.001) than HCM patients. No differences in creatine kinase isoenzyme, TG, and CHOL were found between the CA and HCM groups.

### 3.2. Myocardial Strain Analysis in CA and HCM Patients

The characteristics of RV myocardial function and strain parameters of patients and normal controls are shown in Table 2. In contrast to normal controls, CA and HCM patients showed lower RVEF and RV strain parameters in all three directions (all *p* < 0.05). Similar to the RVEF and RV strain results, the RVT of CA (7.8 ± 2.1 vs 4.0 ± 1.1, *p* < 0.001) and HCM patients (5.9 ± 1.3 vs 4.0 ± 1.1, *p* < 0.001) were both significantly higher than that of the healthy controls. More importantly, patients with CA had higher RVT (7.8 ± 2.1 vs 5.9 ± 1.3, *p* < 0.001) and lower RV-GRPS (12.1 ± 6.9 vs 23.5 ± 12.1, *p* < 0.001), RV-GCPS (−3.4 ± 2.2 vs −5.6 ± 3.5, *p* < 0.001), and RV-GLPS (−4.6 ± 2.3 vs −11.1 ± 4.9, *p* < 0.001) than HCM patients.

Furthermore, in HCM and CA patients with preserved RVEF, the RV radial (15.7 ± 6.3 vs 25.0 ± 9.4, *p* < 0.001), circumferential (−3.3 ± 1.7 vs −6.0 ± 3.7, *p* < 0.001), and longitudinal (−5.5 ± 1.5 vs 11.5 ± 3.5, *p* < 0.001) stain in CA patients were significantly reduced than HCM patients. Moreover, the RVT of CA patients was also thicker than that of HCM patients (7.5 ± 2.1 vs 5.6 ± 1.4, *p* < 0.001) (Table 3).

As shown in Table 4, RV hypertrophy was present in 42 (70%) patients with CA and 23 (38%) patients with HCM. In patients with RV hypertrophy, CA patients had significantly lower RVEF (38.3 ± 15.7 vs 49.1 ± 14.5, *p* = 0.002), RVT (8.7 ± 1.5 vs 7.0 ± 0.8, *p* = 0.001), RV-GRPS (10.5 ± 6.6 vs 22.8 ± 11.9, *p* < 0.001), RV-GCPS (−3.1 ± 2.2 vs −6.4 ± 4.2, *p* < 0.001), and RV-GLPS (−4.1 ± 2.1 vs −11.5 ± 5.1, *p* < 0.001) than HCM patients. Similarly, CA patients without RV hypertrophy had lower RV-GRPS (15.7 ± 6.1 vs 23.9 ± 12.1, *p* = 0.021) and RV-GLPS (−5.8 ± 2.4 vs −10.9 ± 4.8, *p* < 0.001) than HCM patients without RV hypertrophy. No differences were found in RVEF (49.9 ± 16.5 vs 53.3 ± 12.7, *p* = 0.102), RVT (5.5 ± 1.3 vs 5.1 ± 1.0, *p* = 0.120), and RV-GCPS (−3.4 ± 1.8 vs −5.1 ± 3.1, *p* = 0.061) between CA and HCM patients without RV hypertrophy.

### 3.3. Differences in Diagnostic Performance for Detecting CA or HCM

The diagnostic performances of the RV parameters are displayed in Table 5. After calculating the sensitivity, specificity, areas under the curve (AUCs), and cutoff values, we found that the RV parameters, including the RVEF, RVT, and RV strain parameters, had AUCs of 0.68–0.92 for distinguishing CA from HCM.

**Figure 2:**
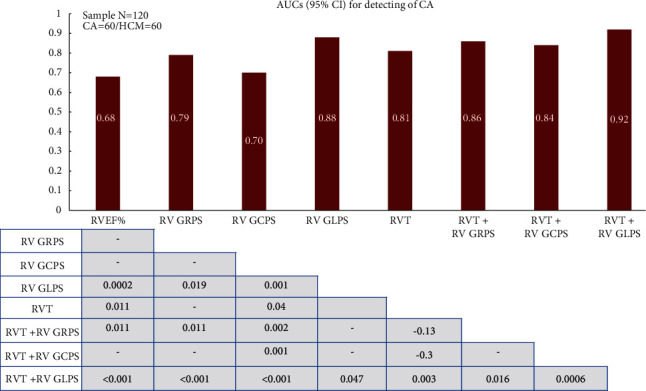
Bar graph showing the areas under the curve (AUCs) and 95% confidence intervals (CIs) as a measure of the diagnostic performance of various right ventricular (RV) parameters for detecting cardiac amyloidosis. The respective cutoff values are represented in Table 4. The cross-table shows the *p* values of the pairwise comparisons of the AUC values. Right ventricular thickness (RVT) combined with RV global longitudinal peak strain (GLPS) showed a significantly larger AUC than the other RV parameters (all *p* < 0.05).

**Figure 3:**
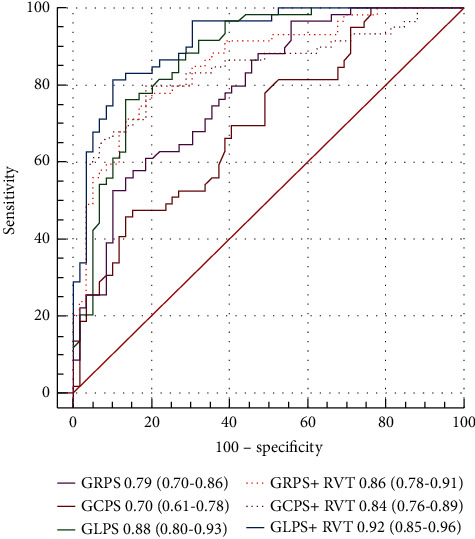
Receiver operating characteristic curves of different right ventricular (RV) cardiac magnetic resonance indices for distinguishing cardiac amyloidosis and hypertrophic cardiomyopathy. Right ventricular thickness (RVT) combined with RV global longitudinal peak strain (GLPS) represent the largest area under the curve (AUC = 0.92, 95% CI 0.85–0.96). Numbers represent areas under the curve and 95% confidence intervals.

Figure 2 shows the comparison of the AUCs of RV parameters for detecting CA. Overall, the RV parameters, especially RV-GLPS combined with RVT, showed the largest AUCs (AUC 0.92, 95% CI 0.85–0.96, *p* = 0.0001) and balanced high sensitivity (sensitivity 81.4%, 95% CI 69.1–90.3) and specificity (specificity 89.8%, 95% CI 79.2–96.1). In contrast, traditional RV parameters such as RVEF showed a low diagnostic efficiency (AUC 0.68, 95% CI: 0.59 to 0.76, *p* = 0.0003). The receiver operating characteristic curves of different RV deformation indices for distinguishing between CA and HCM are shown in Figure 3.

### 3.4. Intra- and Interobserver Variability

**Figure 4:**
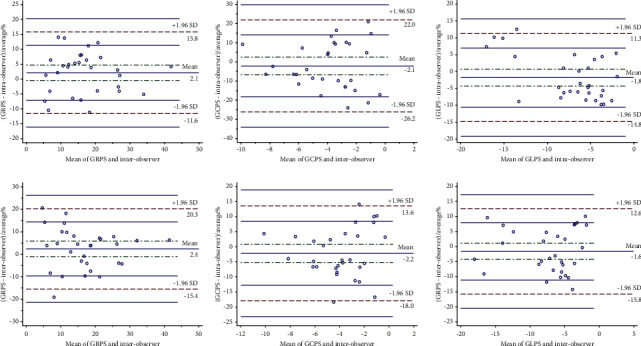
Intra- and interobserver consistency analyses for right ventricular (RV) global radial peak strain (GRPS), circumferential peak strain (GCPS), and longitudinal peak strain (GLPS). The agreements of the RV strain parameters are shown as the percentage mean bias ± SD and 95% limits. The percentage mean bias of the intraobserver agreement was between −1.8%∼2.1% and that of the interobserver agreement was −2.2%∼2.4%.

As shown in Figure 4, the average% of the difference of all measurement results is below 2.4, and most points fall within the 95% consistency limit. Among them, all points of intraobserver of RV-GCPS, RV-GRPS, and interobserver of RV-GLPS fall within the 95% consistency limit. 1 (3.3%) point of intraobserver of RV-GLPS did not fall within the 95% consistency limit. 2 (6.7%) point of interobserver of RV-GRPS and RV-GCPS did not fall within the 95% consistency limit.

## 4. Discussion

Since the prognosis and therapeutic options greatly differ between diseases, the differentiation between CA and HCM has always been a difficult problem in clinical practice. In the present study, we find that the degree of RVT was more severe in CA patients and the RV deformation derived from the CMR-TT technique showed a more significant decline in CA patients than in HCM patients. We further demonstrated that RV-GLPS combined with RVT showed the largest diagnostic accuracy (AUC = 0.92) for distinguishing CA from HCM, showing that the difference in RV plays an important role in the identification of these two diseases.

Cardiac magnetic resonance (CMR) late gadolinium enhancement (LGE) is applied widely for the diagnosis of patients with CA. Additionally, other tissue characterization techniques such as T1-mapping, both native and with measurement of extracellular volume fraction also valuable tool for evaluation of CA [18]. However, different machines and renal failure make it difficult to diagnose CA. Bellavia et al. determined that RVT was more severe in advanced CA patients, and Doppler myocardial imaging measures of the RV can identify early impairment of cardiac function or stratify risk of death in CA patients [19]. Besides Doppler myocardial imaging, noncontrast CMR-TT based on routine cine images also could help evaluate the severity of myocardial deformation from various diseases with high spatial resolution [11, 20–23]. A recent literature also proves that CMR-TT is a reliable method for distinguishing between CA and HCM without administration of gadolinium-contrast [24]. Their study showed no significant differences between AUCs for the LGE pattern (0.994), LV GRPS (0.898), and GCPS (0.880) (all *p* > 0.109). The difference is that they compare the strain of LV; however, we compared the strain of RV. The study of Reddy et al. [25] proved that CMR can be a potent tool for accurate functional assessment of strain and strain rates involving both LV and RV for CA patients, including GRPS (39.7 ± 3.5 vs 13.6 ± 5.1), GCPS (−18.2 ± 1.5 vs −8.1 ± 1.7), and GLPS (−15.3 ± 0.9 vs −6.6 ± 1.4) of the LV and GRPS (−12.1 ± 1.6 vs −6.9 ± 1.2) and GLPS (−15.7 ± 2.1 vs −8.5 ± 1.4) strain of the RV. Nevertheless, their sample size is only 5, and normal people were used as control. Our results first revealed the difference of right ventricular thickness and strain between CA and HCM with reasonable cases. The application of tissue tracking has led to a deeper understanding of dysfunction processes in CA, both in the LV and RV.

## 5. Limitations

There are some limitations in our study. First, a classification of HCM was not performed, which may cause some deviations when these results are applied for all HCM patients. But thinking of obvious differences in morphology between CA and HCM patients with LV outflow tract obstruction or apical hypertrophy, patients with interventricular septum hypertrophy or LV free wall hypertrophy were particularly difficult to diagnose with CA. Therefore, only patients with interventricular septum hypertrophy or LV free wall hypertrophy were enrolled in our study. Second, CMR sequences that evaluated the changes in histology, such as the late gadolinium delayed enhancement, mapping, or extracellular volume sequences, were not included in the present study because it is difficult to identify in the RV to some extent.

## 6. Conclusions

As a promising method, CMR tissue tracking can be used for the quantitative analysis and early detection of subclinical RV deformation. Right ventricular myocardial thickness and deformation of CA patients were more severe than HCM patients. Most importantly, RV-GLPS combined with RVT presented the largest AUC and balanced a high sensitivity and specificity to differentiate between CA and HCM.

## Figures and Tables

**Table 1:** Baseline characteristics of patients of HCM and CA patients.

Parameters	Control subjects (*n* = 30)	HCM (*n* = 60)	CA (*n* = 60)	*p* value (CA vs HCM)
Age	54 ± 12	55 ± 14	58 ± 10	0.676
Male (%)	17 (57%)	27 (45%)	33 (55%)	0.795
Body mass index, kg/m^2^	21.81 ± 1.85	23 ± 2.87	22 ± 3.09	0.318
Myocardial infraction	0 (0%)	0 (0%)	0 (0%)	—
Hypertension	0 (0%)	2 (3%)	3 (5%)	—
Diabetes	0 (0%)	2 (3%)	2 (3%)	—
Systolic pressure, mmHg	121.7 ± 83.6	122 ± 15	113 ± 18	0.036
Diastolic pressure, mmHg	78.9 ± 12.9	72 ± 10	73 ± 13	0.675
Myoglobin	—	31.93 (25.04–45.55)	50.96 (34.46–80.04)	0.005
Creatine kinase isoenzyme	—	3.27 (2.56–6.01)	4.35 (2.33–6.46)	0.086
NT-proBNP, pg/ml	203 ± 105.4	1513 (656–3516) ^*∗∗∗*^	5803.5 (2533–13969) ^*∗∗∗*^	0.001
Troponin *T*	0.7 ± 0.32	17.3 (11.9–45.3) ^*∗∗∗*^	91.9 (53.85–223.45) ^*∗∗∗*^	0.001
TG	—	1.22 (0.89–2.06)	1.29 (0.89–1.75)	0.856
CHOL	—	4.27 ± 1.61	4.09 ± 1.08	0.546

CA, cardiac amyloidosis; HCM, hypertrophic cardiomyopathy; NT-proBNP, N-terminal pro-brain natriuretic peptide; TG, triglyceride; CHOL, cholesterol. ^*∗*^*p* < 0.05, ^*∗∗*^*p* < 0.01, and ^*∗∗∗*^*p* < 0.001 versus controls.

**Table 2:** Cardiac function and myocardial strain in CA and HCM patients.

Parameters	Normal controls (*n* = 30)	HCM (*n* = 60)	CA (*n* = 60)	*p* value (CA vs HCM)
RVEF%	57.1 ± 9.1	51.6 ± 13.5 ^*∗*^	41.5 ± 16.5^∗∗∗^	<0.001
RVEDV-index (ml/m^2^)	60.1 ± 16.4	53.7 ± 11.4 ^*∗*^	58.3 ± 19.9	0.556
RVESV-index (ml/m^2^)	25.3 ± 7.6	25.9 ± 8.9	34.4 ± 16.3^∗∗^	0.001

*Radical*				
RV-PS (%)	31.3 ± 7.6	23.5 ± 12.1^∗∗^	12.1 ± 6.9^∗∗∗^	<0.001
RV-PSSR (1/s)	2.7 ± 1.5	1.9 ± 2.4	1.2 ± 1.2^∗∗∗^	0.097
RV-PDSR (1/s)	−2.7 ± 1.5	−1.5 ± 0.8^∗∗∗^	−1.1 ± 0.8^∗∗∗^	0.079

*Circumferential*				
RV-PS (%)	−13.7 ± 2.5	−5.6 ± 3.5^∗∗∗^	−3.4 ± 2.2 ^*∗∗∗*^	<0.001
RV-PSSR (1/s)	−0.7 ± 0.4	−0.7 ± 0.4	−0.6 ± 0.5 ^*∗∗∗*^	0.086
RV-PDSR (1/s)	0.9 ± 0.5	0.6 ± 0.3	0.5 ± 0.2#	0.048

*Longitudinal*				
RV-PS (%)	−14.23 ± 3.0	11.1 ± 4.9^∗∗∗^	−4.6 ± 2.3^∗∗∗^	<0.001
RV-PSSR (1/s)	−0.6 ± 0.4	−0.7 ± 0.5	−0.9 ± 0.4	0.634
RV-PDSR (1/s)	0.7 ± 0.5	0.8 ± 0.4	0.8 ± 0.4	0.546
RVT (mm)	4.0 ± 1.1	5.9 ± 1.3^∗∗∗^	7.8 ± 2.1^∗∗∗^	<0.001

CA, cardiac amyloidosis; HCM, hypertrophic cardiomyopathy; RVEF, right ventricular ejection fraction; EDV, end-diastolic volume; ESV, end-systolic volume; RV, right ventricular, PS, peak strain; PSSR, peak systolic strain rate; PDSR, peak diastolic strain rate; RVT, right ventricular thickness. ^*∗*^*p* < 0.05, ^*∗∗*^*p* < 0.01, and ^*∗∗∗*^*p* < 0.001 versus controls.

**Table 3:** Cardiac function and myocardial strain in CA and HCM patients with preserved RVEF.

Parameters	HCM (*n* = 40)	CA (*n* = 28)	*p* value
RVEF	58.6 ± 8.2	55.8 ± 7.6	0.166
RVEDV-index (ml/m^2^)	55.2 ± 10.0	56.2 ± 10.1	0.795
RVESV-index (ml/m^2^)	22.7 ± 6.2	24.7 ± 9.7	0.318

*Radical*			
RV-PS (%)	25.0 ± 9.4	15.7 ± 6.3	<0.001
RV-PSSR (1/s)	2.1 ± 0.9	1.2 ± 0.4	0.111
RV-PDSR (1/s)	−1.6 ± 0.4	−1.5 ± 0.6	0.778

*Circumferential*			
RV-PS (%)	−6.0 ± 3.7	−3.3 ± 1.7	<0.001
RV-PSSR (1/s)	−0.7 ± 0.3	−0.5 ± 0.2	0.041
RV-PDSR (1/s)	0.6 ± 0.3	0.5 ± 0.2	0.107

*Longitudinal*			
RV-PS (%)	−11.5 ± 3.5	−5.5 ± 1.5	<0.001
RV-PSSR (1/s)	−0.8 ± 0.3	−0.9 ± 0.4	0.554
RV-PDSR (1/s)	0.9 ± 0.3	0.8 ± 0.3	0.621
RVT (mm)	5.6 ± 1.4	7.5 ± 2.1	<0.001

**Table 4:** Cardiac function and myocardial strain in CA and HCM patients with or without RV hypertrophy.

Parameters	RV hypertrophy			Without RV hypertrophy		
	HCM (*n* = 23)	CA (*n* = 42)	*p* value	HCM (*n* = 37)	CA (*n* = 18)	*p* value
RVEF%	49.1 ± 14.5	38.3 ± 15.7	0.002	53.3 ± 12.7	49.9 ± 16.5	0.102
RVEDV-index (ml/m^2^)	56.1 ± 12.5	61.2 ± 18.8	0.065	52.1 ± 10.6	50.9 ± 22.2	0.165
RVESV-index (ml/m^2^)	28.4 ± 9.1	38.4 ± 16.9	0.045	24.3 ± 8.5	24.1 ± 9.2	0.096

*Radical*						
RV-GPS (%)	22.8 ± 11.9	10.5 ± 6.6	<0.001	23.9 ± 12.2	15.7 ± 6.1	0.021
RV-PSSR (1/s)	1.6 ± 0.7	1.2 ± 1.0	0.157	2.1 ± 0.3	1.2 ± 0.5	0.197
RV-PDSR (1/s)	−1.4 ± 0.8	−1.0 ± 0.7	0.150	−1.6 ± 0.9	−1.4 ± 0.7	0.443

*Circumferential*						
RV-GPS (%)	−6.4 ± 4.2	−3.1 ± 2.2	<0.001	−5.1 ± 3.1	−3.4 ± 1.8	0.061
RV-PSSR (1/s)	−0.7 ± 0.2	−0.6 ± 0.3	0.538	−0.7 ± 0.5	−0.5 ± 0.3	0.246
RV-PDSR (1/s)	0.6 ± 0.3	0.5 ± 0.2	0.375	0.6 ± 0.3	0.6 ± 0.3	0.421

*Longitudinal*						
RV-GPS (%)	−11.5 ± 5.1	−4.1 ± 2.1	<0.001	−10.9 ± 4.8	−5.8 ± 2.4	<0.001
RV-PSSR (1/s)	−0.6 ± 0.5	−0.8 ± 0.5	0.159	−0.9 ± 0.4	−0.9 ± 0.3	0.564
RV-PDSR (1/s)	0.8 ± 0.4	0.8 ± 0.4	0.638	0.8 ± 0.4	0.9 ± 0.4	0.954
RVT (mm)	7.0 ± 0.8	8.7 ± 1.5	0.001	5.1 ± 1.0	5.5 ± 1.3	0.120

CA, cardiac amyloidosis; HCM, hypertrophic cardiomyopathy; RVEF, right ventricular ejection fraction; EDV, end-diastolic volume; ESV, end-systolic volume; RV, right ventricular; PS, peak strain; PSSR, peak systolic strain rate; PDSR, peak diastolic strain rate; RVT, right ventricular thickness.

**Table 5:** Receiver operating characteristic curves of different CMR indices for the distinction between CA and HCM.

Variables	AUC	95% CI	*p* value	Cutoff	Sens, %	95% CI	Spec, %	95% CI	+LR	−LR	+PV	−PV
RVEF%	0.68	0.59–0.76	0.0003	≤39.86	52.54	39.1–65.7	79.66	67.2–89.0	2.58	0.6	72.10	62.70
RV-GRPS	0.79	0.70–0.86	0.0001	≤10.19	52.54	39.1–65.7	89.83	79.2–96.1	5.17	0.53	83.80	65.40
RV-GCPS	0.70	0.61–0.78	0.0001	≥−2.63	45.76	32.7–59.2	86.44	75.0–93.9	3.38	0.63	77.10	61.40
RV-GLPS	0.88	0.80–0.93	0.0001	≥−5.87	76.27	63.4–86.4	86.40	75.0–93.9	5.63	0.27	84.90	78.50
RVT	0.81	0.73–0.89	0.0001	≥6.70	73.33	60.3–83.9	83.33	71.5–91.7	4.4	0.32	81.50	75.80
RVT + RV-GRPS	0.86	0.78–0.91	0.0001	≤0.38	71.20	57.9–82.2	86.40	75.0–93.9	5.25	0.33	84.00	75.00
RVT + RV-GCPS	0.84	0.76–0.89	0.0001	≤0.47	79.70	67.2–89.0	81.40	69.1–90.3	4.27	0.25	81.00	80.00
RVT + RV-GLPS	0.92	0.85–0.96	0.0001	≤0.36	81.36	69.1–90.3	89.83	79.2–96.1	8.00	0.21	88.90	82.80

CA, cardiac amyloidosis; HCM, hypertrophic cardiomyopathy; CMR, cardiac magnetic resonance; RVEF, right ventricular ejection fraction; GCPS, global circumferential peak strain; GLPS, global longitudinal peak strain; GRPS, global radial peak strain; RVT, right ventricular thickness; AUC, area under the curve; Sens, sensibility; Spec, specificity; +LR, positive likelihood ratio; −LR, negative likelihood ratio; +PV, positive predictive value; −PV, negative predictive value.

## Data Availability

The data used to support the findings of this study are available from the corresponding author upon request.

## References

[1] Dubrey S., Cha K., Anderson J. (1998). The clinical features of immunoglobulin light-chain (AL) amyloidosis with heart involvement. *QJM*.

[2] Maron B. J., Maron M. S. (2013). Hypertrophic cardiomyopathy. *The Lancet*.

[3] Efthimiadis G. K., Pagourelias E. D., Hadjimiltiades S., Meditskou S., Karvounis H., McKenna W. J. (2015). Feasibility and significance of preclinical diagnosis in hypertrophic cardiomyopathy. *Cardiology in Review*.

[4] Pagourelias E. D., Mirea O., Duchenne J. (2017). Echo parameters for differential diagnosis in cardiac amyloidosis: a head-to-head comparison of deformation and nondeformation parameters. *Circulation. Cardiovascular imaging*.

[5] Pagourelias E. D., Mirea O., Vovas G. (2019). Relation of regional myocardial structure and function in hypertrophic cardiomyopathy and amyloidois: a combined two-dimensional speckle tracking and cardiovascular magnetic resonance analysis. *European Heart Journal-Cardiovascular Imaging*.

[6] Pagourelias E. D., Duchenne J., Mirea O. (2016). The relation of ejection fraction and global longitudinal strain in amyloidosis: implications for differential diagnosis. *Journal of the American College of Cardiology: Cardiovascular Imaging*.

[7] Arvidsson S., Henein M. Y., Wikström G., Suhr O. B., Lindqvist P. (2018). Right ventricular involvement in transthyretin amyloidosis. *Amyloid: International Journal of Experimental & Clinical Investigation*.

[8] Claus P., Omar A. M. S., Pedrizzetti G., Sengupta P. P., Nagel E. (2015). Tissue tracking Technology for assessing cardiac mechanics. *Journal of the American College of Cardiology: Cardiovascular Imaging*.

[9] Winther S., Williams L. K., Keir M. (2019). Cardiovascular magnetic resonance provides evidence of abnormal myocardial strain and primary cardiomyopathy in marfan syndrome. *Journal of Computer Assisted Tomography*.

[10] Tahir E., Starekova J., Muellerleile K. (2019). Impact of myocardial fibrosis on left ventricular function evaluated by feature-tracking myocardial strain cardiac magnetic resonance in competitive male triathletes with normal ejection fraction. *Circulation Journal*.

[11] Gatti M., Palmisano A., Faletti R. (2019). Two-dimensional and three-dimensional cardiac magnetic resonance feature-tracking myocardial strain analysis in acute myocarditis patients with preserved ejection fraction. *The International Journal of Cardiovascular Imaging*.

[12] Aschauer S., Kammerlander A. A., Zotter-Tufaro C. (2016). The right heart in heart failure with preserved ejection fraction: insights from cardiac magnetic resonance imaging and invasive haemodynamics. *European Journal of Heart Failure*.

[13] Jaccard A., Moreau P., Leblond V. (2007). High-dose melphalan versus melphalan plus dexamethasone for AL amyloidosis. *New England Journal of Medicine*.

[14] Gertz M., Comenzo R., Falk R. (2010). Definition of organ involvement and treatment response in immunoglobulin light chain amyloidosis (AL): a consensus opinion from the 10th International Symposium on Amyloid and Amyloidosis. *American Journal of Hematology*.

[15] Elliott P. M., Elliott P. M., Anastasakis A. (2014). 2014 ESC guidelines on diagnosis and management of hypertrophic cardiomyopathy: the task force for the diagnosis and management of hypertrophic cardiomyopathy of the european society of cardiology (ESC). *European Heart Journal*.

[16] Foale R., Nihoyannopoulos P., McKenna W. (1986). Echocardiographic measurement of the normal adult right ventricle. *Heart*.

[17] DeLong E. R., DeLong D. M., Clarke-Pearson D. L. (1988). Comparing the areas under two or more correlated receiver operating characteristic curves: a nonparametric approach. *Biometrics*.

[18] Patel A. R., Kramer C. M. (2017). Role of cardiac magnetic resonance in the diagnosis and prognosis of nonischemic cardiomyopathy. *Journal of the American College of Cardiology: Cardiovascular Imaging*.

[19] Bellavia D., Pellikka P. A., Dispenzieri A. (2012). Comparison of right ventricular longitudinal strain imaging, tricuspid annular plane systolic excursion, and cardiac biomarkers for early diagnosis of cardiac involvement and risk stratification in primary systematic (AL) amyloidosis: a 5-year cohort study. *European Heart Journal - Cardiovascular Imaging*.

[20] Bourfiss M., Vigneault D. M., Aliyari Ghasebeh M. (2017). Feature tracking CMR reveals abnormal strain in preclinical arrhythmogenic right ventricular dysplasia/cardiomyopathy: a multisoftware feasibility and clinical implementation study. *Journal of Cardiovascular Magnetic Resonance*.

[21] Vigneault D. M., te Riele A. S. J. M., James C. A. (2016). Right ventricular strain by MR quantitatively identifies regional dysfunction in patients with arrhythmogenic right ventricular cardiomyopathy. *Journal of Magnetic Resonance Imaging*.

[22] Gavara J., Rodriguez-Palomares J. F., Valente F. (2018). Prognostic value of strain by tissue tracking cardiac magnetic resonance after ST-segment elevation myocardial infarction. *Journal of the American College of Cardiology: Cardiovascular Imaging*.

[23] O’Hanlon R., Pennell D. J. (2009). Cardiovascular magnetic resonance in the evaluation of hypertrophic and infiltrative cardiomyopathies. *Heart Failure Clinics*.

[24] Jung H. N., Kim S. M., Lee J. H. (2020). Comparison of tissue tracking assessment by cardiovascular magnetic resonance for cardiac amyloidosis and hypertrophic cardiomyopathy. *Acta Radiologica*.

[25] Reddy A., Singh V., Karthikeyan B. (2021). Biventricular strain imaging with cardiac MRI in genotyped and histology validated amyloid cardiomyopathy. *Cardiogenetics*.

